# Taxonomic and functional diversity increase the aesthetic value of coralligenous reefs

**DOI:** 10.1038/srep34229

**Published:** 2016-09-28

**Authors:** Anne-Sophie Tribot, Nicolas Mouquet, Sébastien Villéger, Michel Raymond, Fabrice Hoff, Pierre Boissery, Florian Holon, Julie Deter

**Affiliations:** 1 Institut des Sciences de l’Evolution (ISEM)–UMR 5554 CNRS-UM-IRD, Université de Montpellier, Place Eugène Bataillon, CC 065, FR-34095 Montpellier Cedex 5, France; 2 MARBEC (MARine Biodiversity Exploitation and Conservation), UMR 9190 IRD-CNRS-UM-IFREMER, Université de Montpellier, Place Eugène Bataillon, CC 093, FR-34095 Montpellier Cedex 5, France; 3 Ludiformation, 5 rue de l’Aiglon, 34000 Montpellier, France; 4 Agence de l’Eau Rhône-Méditerranée-Corse, Délégation de Marseille-Immeuble le Noailles, 62 La Canebière-13001 Marseille, France; 5 Andromède Océanologie, 7 place Cassan, 34280 Carnon, France

## Abstract

The aesthetic value of landscapes contributes to human well-being. However, studies which have investigated the link between biodiversity and ecosystem services have not taken aesthetic value into account. In this study we evaluated how the aesthetics of coralligenous reefs, a key marine ecosystem in the Mediterranean, is perceived by the general public and how aesthetic preferences are related to biodiversity facets (taxonomic, phylogenetic and functional diversities). We performed both biodiversity measures and online-surveys of aesthetic perception on photographic quadrats sampled along the French Mediterranean coast. Our results show that species richness and functional richness have a significant positive effect on aesthetic value. Most of the ecological literature, exploring the relationship between biodiversity and ecosystem functioning and service has focused so far on ‘economical’ aspects of biodiversity (provision or regulation). Our results illustrate that cultural facets, such as ‘beauty’, should also be central in our motivations to preserve ecological diversity.

Natural landscapes, defined as an association between species and physical elements, provide valuable ecosystem services to humanity^1^,^2^, including cultural ecosystem services through their aesthetic value^3^. Aesthetic value is an immediate and unconscious phenomenon partly resulting from cognitive mechanisms^4^ which regulates and controls emotions^5^,^6^. It contributes to human well-being through cultural enrichment, cognitive development, reflection and/or recreation experiences^1^. Due to this central role in our relationship with nature, aesthetic value should be considered as a natural resource and hence be included in conservation planning^7^. Biodiversity is a central element of this aesthetic value and yet it has not been directly linked to its perception by human beings. Indeed, most of the studies that have investigated the effects of biodiversity on ecosystem services have focused so far on ‘economical’ aspects such as provisioning or regulation services^8^,^9^, while the links between aesthetic value and biodiversity has rarely been explored.

Biodiversity is made up of three main components that complement ecosystem functioning and ecosystem services: (i) taxonomic diversity (TD) that only accounts for species composition and abundance^10^; (ii) phylogenetic diversity (PD) that accounts for the evolutionary history of species^11^,^12^,^13^ and (iii) functional diversity that accounts for the ecological traits of species^12^. A relationship between these different facets of biodiversity and aesthetic value might be expected. Indeed, an important hypothesis in environmental aesthetics is that human responses to ecosystem characteristics have evolutionary origins^14^,^15^,^16^,^17^: humans seek beneficial habitats, with functional features and processes essential to their survival and well-being^18^. Thereby, people generally tend to interpret their aesthetic preferences of landscapes alongside its ecological quality.

Many studies have been carried out to assess the aesthetic value of landscapes since the 1970s^19^. These studies observed a positive correlation between aesthetic preferences and ‘ecological values’ of the landscapes. However, the ecological value in these studies was defined as ‘landscape’ diversity which includes only physical metrics such as structural diversity of patterns, diversity of land cover, patch diversity, richness and evenness. Very few studies have explored the link between biodiversity and human preferences for landscapes and most of them have used indirect evaluations. For example Hale *et al*.^20^ measured bird species diversity as a function of changes in housing density and found that the diversity of avian species significantly increased in locations that adopted aesthetic landscape planning.

Contrary to terrestrial ecosystems, there has been few assessment of the aesthetic value of underwater ecosystems although they serve as recreational areas for millions of people through diving and are highly threatened by human activities^21^. Their interest is recognized by the general public, for example by the ‘Citizens’ Network for Observation of Marine Biodiversity’ in Europe (http://www.comber.hcmr.gr^22^), the ‘Mediterranean Citizen Observatory for underwater landscapes’ in France (http://ecorem.fr/medobssub^23^) and the ‘purple octopus’ project in United Kingdom (http://www.purpleoctopus.org^24^). Among marine ecosystems, coralligenous reefs are emblematic assemblages mainly found between 20 and 120 m deep. They are composed of biogenic concretions, primarily produced through the accumulation of encrusting algae which grow at low light levels^25^ and secondarily by bio-constructor animals such as polychaetes, bryozoans and gorgonians. They host more than 1700 species dominated by encrusting algae, suspension feeders, borers or soft-bottom fauna^26^ (Fig. 1a). These assemblages are similar to tropical reefs in terms of species richness and abundance^26^, placing them among one of the most diversified ecosystems in the Mediterranean sea.

Here, we used an extensive database detailing coralligenous assemblages sampled along the French Mediterranean coast (113 stations) and questionnaires (1260 responses) to assess how the three main facets of biodiversity (taxonomic, phylogenetic and functional) are related to the aesthetic value of these assemblages.

## Results

### Effect of bio-physical parameters on aesthetic preferences

#### Sample profile and aesthetic score distribution

The online survey was completed by 1260 people: 60% were women. The median age was 30 years; the most represented age range was 18–30 years (46%). Managers and engineers were substantially overrepresented (50%), as well ‘Environment’ and ‘Biology’ professional sectors (33% and 29%, respectively). Around half of the respondents lived more than 20 km from the coast (Supplementary Figure 1).

On the basis of the 1260 responses, the aesthetic scores of the photos ranged from 1083 to 1946. The distribution of scores were significantly different from a random choice (Supplementary Figure 2, p-value < 0.001). There was no difference between the aesthetic score distribution between the whole sample and each of the socio-economical categories (all Kolmogorov-Smirnov p-values < 0.001).

#### Diversity and aesthetic preferences

Significant positive correlations were observed between the aesthetic scores of photos and diversity indices (Fig. 2a). The highest correlation was obtained for TD indices (number of species, Shannon and Simpson) followed by PD and standardized FRic (Fig. 2a). Other functional diversity indices were not significantly correlated with aesthetic scores. The best linear models fitted were for species richness and for the Shannon index (Fig. 2a,b). We found a correlation between PD and TD (Spearman’s rho = 0.515, p-value < 0.001) and between FRic and TD (Spearman’s rho = 0.834, p-value < 0.001). To correct for this effect, we performed linear models between PD and TD (R^2^ = 0.605, p-value < 0.001) and between FRic and TD (R^2^ = 0.274, p-value < 0.001). We correlated the aesthetic scores with the residuals of these relationships and still found a positive correlation between corrected FRic and aesthetic scores (Spearman’s rho = 0.171, p-value < 0.01) but not for PD (p-value > 0.05).

We found that the aesthetic scores were negatively correlated with the percentage of non-colonized substratum and the sediment percentage cover (Table 1a, n = 1260). To correct for this effect, we correlated the diversity indices with the residual of this relationship, and still found a significant (but lower) relationship for all of the indices except FEve and FDiv (Table 1a, n = 1260). We also found that the aesthetic scores were positively correlated with the index of Marginal colour heterogeneity for Red, Green, Saturation and Value channels (Table 1a, n = 1260). Note that percentages of non-colonized substratum and sediment cover were negatively correlated with the Marginal colour heterogeneity index for the saturation channel (Table 1a, n = 1260). Finally, we found a positive relationship between the diversity of colours (measured using Shannon’s index) and the aesthetic scores (Spearman’s rho = 0.381, p-value < 0.001).

#### Identification of the key functional traits, colours and species groups

We found that aesthetic score was positively correlated with FRic calculated with only the traits related to aesthetics (Spearman’s rho = 0.353, Table 1b and Supplementary Table 1: classes ‘colour’, ‘protect’, ‘interest’, ‘shape’; n = 1260). We then performed a sensitivity analysis to identify which of these traits had the greatest impact on this relationship (aesthetic importance of removed traits δ, as defined in methods). We found that the most important traits were related to the shape (δ = 0.055, Table 1b, n = 1260), particularly ‘base type’ (encrusting, semi-erect or erect, δ = 0.041, Table 1b, n = 1260). Among the ‘base type’ traits, the coverage of erect species per quadrat was positively correlated with the aesthetic score (Spearman’s rho = 0.345, Table 1b), whereas the coverage of encrusting species was negatively correlated with it (Spearman’s rho = −0.116, Table 1b). Species colour abundances, based on the coverage per photo of species for each colour (e.g. coverage of red species), were not correlated with aesthetic scores (all p-values > 0.05).

Finally, among all of the paraphyletic groups tested, only four had their coverages positively correlated to the aesthetics score (green algae: Spearman’s rho = 0.244, gorgonians and corals: Spearman’s rho = 0.247, sea urchins: Spearman’s rho = 0.119, and bryozoans: Spearman’s rho = 0.127). Within each group, we removed every species and tested for their importance in the original correlation (Supplementary Figure 3). We found that for green algae, the difference was higher when *Flabellia petiolata* was removed (δ = 0.100, Supplementary Figure 3). For gorgonians and corals (and similar species), the difference was higher when *Eunicella cavolini* (δ = 0.041, Supplementary Figure 3), *Leptopsammia pruvoti* (δ = 0.026, Supplementary Figure 3) and *Paramuricea clavata* (δ = 0.014, Supplementary Figure 3) were removed. Conversely, the presence of ‘*Hydrozoa*’ decreased the correlation between the coverage of species and the aesthetic score (δ = −0.05, Supplementary Figure 3). Among bryozoans, the difference was higher when *Myriapora truncata* was removed (δ = 0.016, Supplementary Figure 3).

#### Observer’s justifications

The coverages of observed colours were correlated with observer preferences (Spearman’s rho = 0.761, p-value < 0.05). Purple, red and orange were the most frequently preferred colours (Fig. 3a). When compared with the bisector of the observed coverages/preferred frequencies, purple and red were more often preferred and orange less often preferred than expected based on their observed coverages.

Observed coverages for each species group were correlated with observer preferences (Spearman’s rho = 0.857, p-value < 0.05). Corals and gorgonians, red algae, and sponges were the most frequently preferred groups (Fig. 3b). When compared with the bisector of the observed coverages/preferred frequencies, gorgonians and corals were more often preferred and sponges and red algae less often preferred than expected based on their observed coverages. Finally, observers most often answered that colour diversity; colour intensity; colour brightness and high contrast contributed the most to their preferences (Supplementary Figure 4). The diversity of forms and reliefs were also important. With regards to ‘feelings’, the main arguments were curiosity, serenity, and similarity to the ‘seabed reference’ (‘This photo matches with my vision of the seabed’, Fig. 4b).

#### Mapping

We found that biodiversity measured in the 3 quadrats used to test aesthetic score provides a relevant proxy of the biodiversity present at each station (i.e. on the set of 30 quadrats recorded in each station, Supplementary Figure 5). We thus map on Fig. 5 estimation of the different facets of coralligenous assemblages biodiversity, including aesthetic value, along the French Mediterranean coast. Most beautiful stations were found in Port-Cros and Calanques National Parks (next to Hyeres and Marseille, respectively), off Saint-Florent (northern Corsica) and along the Corsican west coast (Fig. 5a). The least aesthetic stations were primarily in the Gulf of Lion, off Nice (northern Mediterranean) and Ajaccio (southern Corsica). These areas corresponded to hotspots of species diversity (TD, Fig. 5b) and Functional Richness (FRic, Fig. 5c) especially for the Port-Cros National Park. However, the FRic map did not match with the aesthetic map for the Calanques National Park and for the north of Corsica (Fig. 5c).

## Discussion

We found that biodiversity, especially TD and FRic to a lesser extent, were correlated with aesthetic scores. Similarly, the Marginal colour heterogeneity indices calculated for saturation channel (which corresponds to colour intensity) and value channel (which corresponds to colour brightness), were also positively correlated with aesthetic score. Indeed, in our assessment of scenic value at a fine scale, our colour heterogeneity indices can be interpreted as a measure of structural diversity, which is an indicator of scenic beauty^27^,^28^,^29^,^30^,^31^. We did not find any additive effect of PD on aesthetic score than what was already explained by TD. This weak effect of PD illustrate that species with the highest aesthetic interest (i.e. corals and gorgonians) belong to the same phylogenetic group.

Regarding functional diversity, after correcting for TD, FRic was still correlated with aesthetic score. This suggests that the range of combinations of functional traits plays a role in aesthetic preferences. However, humans do not seem to perceive the other functional dimensions such as functional evenness (FEve) or divergence in the abundance of traits (FDiv) as aesthetic factors. We hypothesise that during the observation, attention was focused on erect species, bright and varied colours, especially red and purple, which are the species with the most original combinations of traits values.

We found that the percentage cover of sediment and non-colonized substrate has a significant negative impact on aesthetic scores. Moreover, the correlation between aesthetic scores and TD was still significant when we corrected the effects of sediment and non-colonized substrate on aesthetic scores. It has been shown that for terrestrial ecosystems, human aesthetic preferences are related to the search for beneficial habitats, with functional features and processes essential to their ‘survival and well-being’^18^. This effect also applies here; as humans used both aesthetic (like TD) and rejection parameters (such as sediment and non-colonized substrate) to assess the ecological value of this marine landscape.

According to the ecological valence theory of human colour preference^32^, humans prefer colours strongly associated with objects they like (e.g., blue is associated with clear skies and clean water) and dislike colours strongly associated with objects they dislike (e.g., brown is associated with faeces and rotten food). These colour preferences derive from the average person’s affective responses to colour-associated objects that arose from evolutionary selection^33^. People are thus more likely to survive and reproduce successfully if they are attracted to objects whose colours ‘look good’ to them and avoid objects whose colours ‘look bad’ to them, such as sediment and non-colonized substrate^32^.

Here, however we found that purple and red were the most preferred colours, despite their low observed coverages obtained using HSV channels (1% for purple and 13.6% for red vs. 70% for orange). From a psychological point of view, red is often reported to cause excitement and arousal^34^. Counter intuitively, blue and green, that are known to be attractive^33^, were less preferred. This may be explained by their low coverages in the photos (coverages of 0.2% for green, and 0.5% for blue). Indeed, within the circalittoral shelf, where coralligenous reefs grow, chlorophyll organisms (including green algae) are less abundant because of a lack of light, and blue pigments are relatively rare in animal colouration^35^.

We found that an abundance of green algae, corals and gorgonians, sea urchins and some bryozoans were positively correlated with aesthetic scores. Among these groups, some species such as the green algae *Flabellia petiolata* or the gorgonian *Eunicella cavolini* had a very strong influence. After correcting for the observed abundance of these groups, we found that only gorgonians and corals were actually preferred while green algae was less preferred. As managers and engineers were substantially overrepresented, as well ‘Environment’ and ‘Biology’ professional sectors, we fitted a linear model between educated professionals and non-educated professionals preferences frequencies for paraphyletic groups (Supplementary Figure 6). We found that paraphyletic groups preferences for these two groups of people were highly linked (p-value = 2.878 × 10^−06^, R^2^ = 0.975). Corroborating this preference, erect bearing was the functional trait most related to aesthetics. Gorgonians and corals often offer vertical curved lines which is the most powerful aesthetic primitive pictorial that contributes to the perception and preference of visual art^36^. Observer’s preference justifications corroborate this result as they often select “shape” and “colour diversity” as important criteria. Further research would be interesting to test other preference patterns such as, for example, the symmetry of geometric shapes perceptible in seascapes^4^.

This study was carried out using fine-scale photographic quadrats (0.25 m^2^) which was used previously to estimate the diversity of coralligenous assemblages along the French Mediterranean coast^37^. This fine scale might not be sufficiently large to estimate the aesthetic value of entire station where landscape perception becomes the key process for connecting humans with ecological phenomena^3^,^38^. However it was enough to generate a relatively large richness gradient (from 2 species to 19 species) that was needed to link diversity with aesthetic perception in our statistical analysis. The next step will be to evaluate aesthetic preferences at a broader scale combined with exhaustive estimation of biodiversity to fully integrate the ecological metrics of biological diversity within the classical landscape perception literature. This cross scale comparison will also allow evaluating the most relevant scale of human aesthetic perception, i.e. if preference is related to combination of fine scale information or large scale averaging, and how the biotic components are perceived at various organisational scales.

We also acknowledge the limitation of using three (randomly chosen) photographic quadrats to map the distribution of aesthetic scores along the French coast, as well as the limitation of using the kriging method. The main purpose of our study was to evaluate how the aesthetic perception of coralligenous photographic quadrats is related to three main different facets of biodiversity. Our aim here was not to provide an operational map of aesthetic landscape perception but rather to illustrate the potential of mapping this facet of diversity and its potential use by conservation biologists and stakeholders. Map provided in Fig. 5 should be interpreted as an estimation of aesthetic value of Mediterranean coralligenous assemblages, which might be refined and extended to be used in conservation planning.

Our results also suggest that a photo appears to be appreciated if it corresponds to the ideas that the observer has on a seabed (‘this photo matches with my vision of the seabed’, Fig. 4b). This result, however, warrants further investigation into references of the seabed in the collective unconscious. For examples, how much are they related to reality, documentary photos, movies or aquariums? Are they representative of the functionality of marine ecosystems? Further research including a more transactional approach would be useful to assess the effect of this unconscious ‘ideal’ seabed on the perception of aesthetic biodiversity.

## Conclusions

Traditional studies have explored the relationship between biodiversity and ecosystem functioning and economical facets of biodiversity (provision or regulation). Our study focused on ecological ‘beauty’ through the lens of representative parameters of social perceptions. Aesthetic preference was related to the number of species and to the range of their functional traits. These results correlate with actual theories on the evolutionary origins of aesthetic preferences^14^,^16^,^17^, which argue that aesthetic preferences are led by people’s relationship with information and by human survival. Even if humans are only scarcely experiencing the underwater environment during their life, the aesthetic clues used to perceive a potential favourable terrestrial habitat (indicators of ecological value) can potentially be transposed to marine habitats.

The outputs from our study have important implications for the conservation of ecosystems, since aesthetic experience induces a social motivation for ecosystem conservation. Landscape thus appears to be a good vehicle for educating the general public on environmental issues^3^,^38^. However, in order to relate aesthetic preferences to ecological functionality, there still needs to be an understanding of ecological functioning. The main challenge in ecological aesthetics then lies in reconciling the objectives set by ecologists on the basis of rational knowledge, with social expectations based on a subjective image of reality^39^. Education could be a solution to better converge ecology and beauty through the promotion of ‘functional beauty’^18^,^40^ based both on ecological understanding and aesthetic experience.

## Material and Methods

### Sampling of coralligenous assemblages

**Figure 1:**
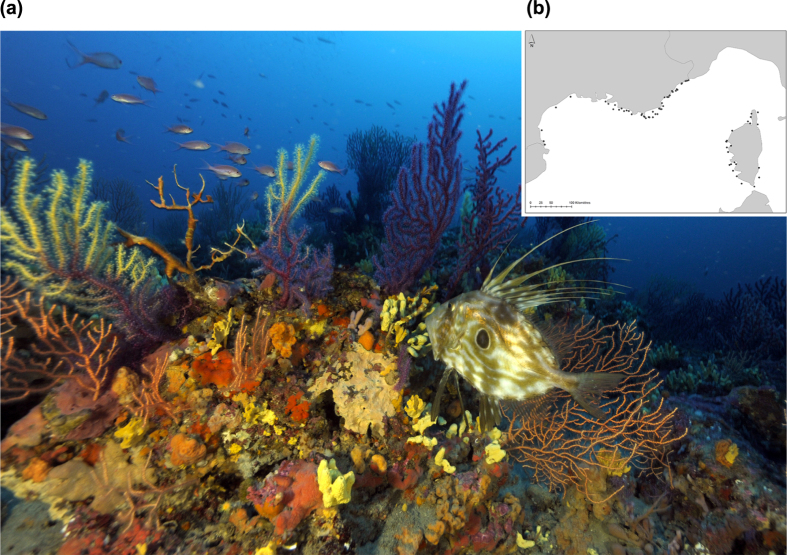
(**a**) Coralligenous assemblage in the Mediterranean. Copyright: Laurent Ballesta for Andromède Océanologie/Agence de l’eau RMC: Campagne RECOR 2011. Photograph taken by Laurent Ballesta. (**b**) Map of the 113 stations sampled in the French Mediterranean. Stations ranged from −20 to −90 m deep. Each station was sampled by three photographic quadrats taken at the same depth. Map generated by using R 3.2.4 2016-03-10 (R: A Language and Environment for Statistical Computing, R Core Team, R Foundation for Statistical Computing, Vienna, Austria (2016) https://www.R-project.org), created by Florian Holon for Andromède Océanologie/Agence de l’Eau RMC.

**Figure 2:**
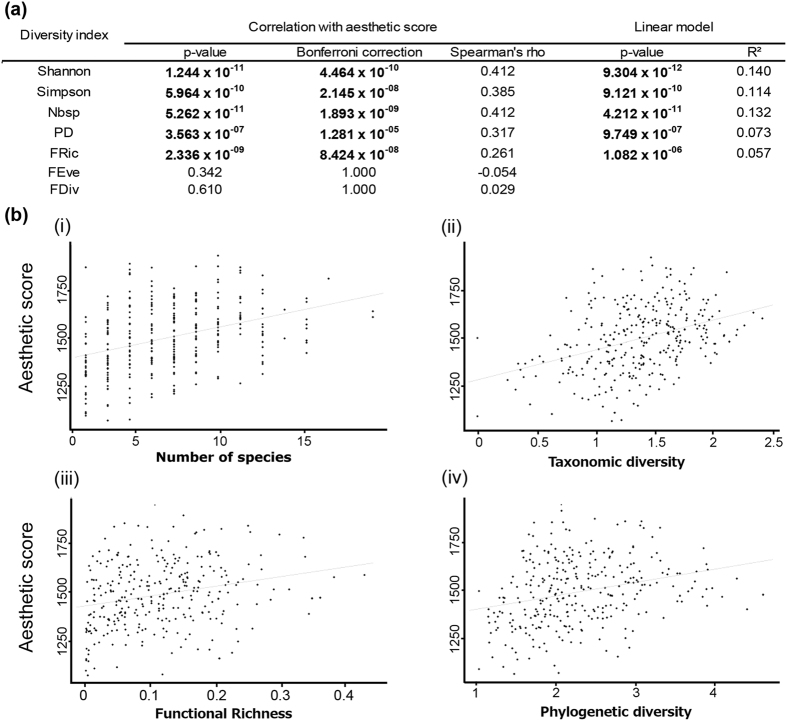
(**a**) Correlation coefficients between the aesthetic scores of photos and diversity indices (n = 1260). Nbsp: number of species. PD: Phylogenetic diversity. FRic: standardized Functional Richness. FEve: Functional evenness. FDiv: Functional divergence. Linear model was not performed for FEve and FDiv because their correlations with aesthetic scores were not significant. Bold values: significant p-values. R^2^ = correlation coefficient. Bonferroni correction = adjusted p-values after a Bonferroni correction. (**b)** Relationship between aesthetic scores and diversity indices. n = 1260. (i) Linear model for aesthetic scores as a function of the number of species per quadrat. R^2^ = 0.132. (ii) Linear model for aesthetic scores as a function of taxonomic diversity measured using Shannon’s index (expressed as equivalent number of species). R^2^ = 0.140. Linear model for aesthetic scores as a function of Simpson’s index is not presented because the relationship is not better than with Shannon’s index. R^2^ = 0.164. (iii) Linear model for aesthetic scores as a function of standardized FRic. R^2^ = 0.057. (iv) Linear model for aesthetic scores as a function of PD. R^2^ = 0.073.

**Figure 3:**
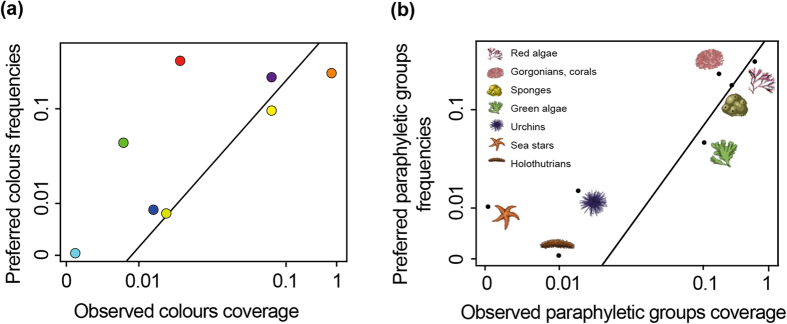
Relationship between observed coverages and preferred frequencies for (**a**) colours and (**b**) species groups. The vertical axis represents the preferred total frequencies and the horizontal axis represents the observed total coverages. Black lines represent the bisectors. N = 338 quadrats. Pictograms provided by DORIS http://doris.ffessm.fr.

We used 338 standardized photographic quadrats (50 by 50 cm) from 113 stations (three quadrats per station) taken along the French Mediterranean coast (Fig. 1b) at a depth between −20 and −90 m during the RECOR program (http://www.observatoire-mer.fr/en/img/recor_en.pdf^41^) which was initiated in 2010 by the French Water Agency and Andromede Oceanology (www.andromede-ocean.com). Each of the three quadrats used for each station were randomly sampled among the 30 quadrats photographed per station by during the RECOR program. Each photo was analysed by a single person using 64 random points via the CPCe 4.1 software ≪ coralligenous assemblages version ≫^42^,^43^. For each quadrat, the percentage of area covered by sediment and non-colonized substratum were measured^37^. Benthic species present in the quadrat were also identified according to the taxonomic nomenclatures of Guiry and Guiry^44^ and Rodriquez-Prieto *et al*.^45^ and classified into recognizable paraphyletic groups: green algae, red algae, brown algae, gorgonians and corals, sponges, tunicates, anemones, sea cucumbers, sea urchins, bryozoans or other cnidarians. Finally the relative abundance of each benthic species was estimated as a coverage among living organisms.

For each quadrat, a Marginal colour heterogeneity index was calculated for each channel of RGB (Red, Green, Blue) and HSV (Hue, Saturation, Value) colour systems^46^. The Marginal colour heterogeneity index quantifies the spatial entropy of the pixel array, scaled between 0 (a single pixel class on the image) and 1 (all pixel values are equally represented). It is calculated using the Shannon entropy equation (1):


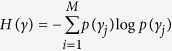


with p (γ) the proportion of pixels in the image that have the value γ and *M* the number of pixel classes.

### Diversity indices

The main purpose of our study was to link the aesthetic perception of coralligenous reefs quadrats to their realized diversity. Thus, for each photographic quadrat, the three main facets of biodiversity were assessed using complementary diversity indices:

- Taxonomic diversity (TD) was assessed using the number of species. Shannon and Simpson indices (based on relative species abundance) were also calculated and converted into effective numbers of species^47^.
- Phylogenetic diversity (PD) was measured by the phylogenetic distance between species. As no molecular sequence data existed for all the studied species, phylogenetic distances were based on species classification data from the literature^37^. Phylogenetic distances between species were then estimated as the distance between a set of tips of the phylogenetic tree. Species distances were estimated for each pair of species in the community. PD was computed using the Rao’s quadratic entropy index given by the equation 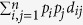 where *p*_*i*_ and *p*_*j*_ are the relative abundance of species and *i, j* and *d*_*ij*_ are the phylogenetic distances between species, converted into effective numbers of species^47^.
- Functional diversity was measured using species functional traits and relative abundance of species. We selected a set of 52 traits that describe complementary facets of coralligenous species such as morphology, feeding, reproduction and defence strategies (Supplementary Table 1 37). Even if some of these traits are not known to have a direct effect on ecosystem functioning, they are all clearly linked to the ecology of the species and thus were considered as ‘functional traits’^48^. Functional traits were classified into two classes: traits related to the ecology of species (e.g. reproduction mode) and traits related to aesthetics (e.g. shape and colour of the species) (Supplementary Table 1). A multidimensional functional space was then built based on the value of the trait for each species^49^ by computing Gower’s distance between species, a Principal Coordinate Analysis on this distance matrix, and selecting the optimal number of axes according to an objective index of a posteriori quality. On the basis of species position in this functional space and the relative abundance of each species in each assemblage we computed three functional diversity indices^50^: Functional Richness (FRic) that quantified the proportion of functional space filled by the community; Functional Evenness (FEve), that quantified the regularity of abundance distribution in the functional space, and Functional Divergence (FDiv) that quantified the distribution of abundance within the portion of functional space filled. These three indices are independent from each other and FEve and FDiv are independent from species richness^50^.

### Questionnaires and photo ranking

**Figure 4:**
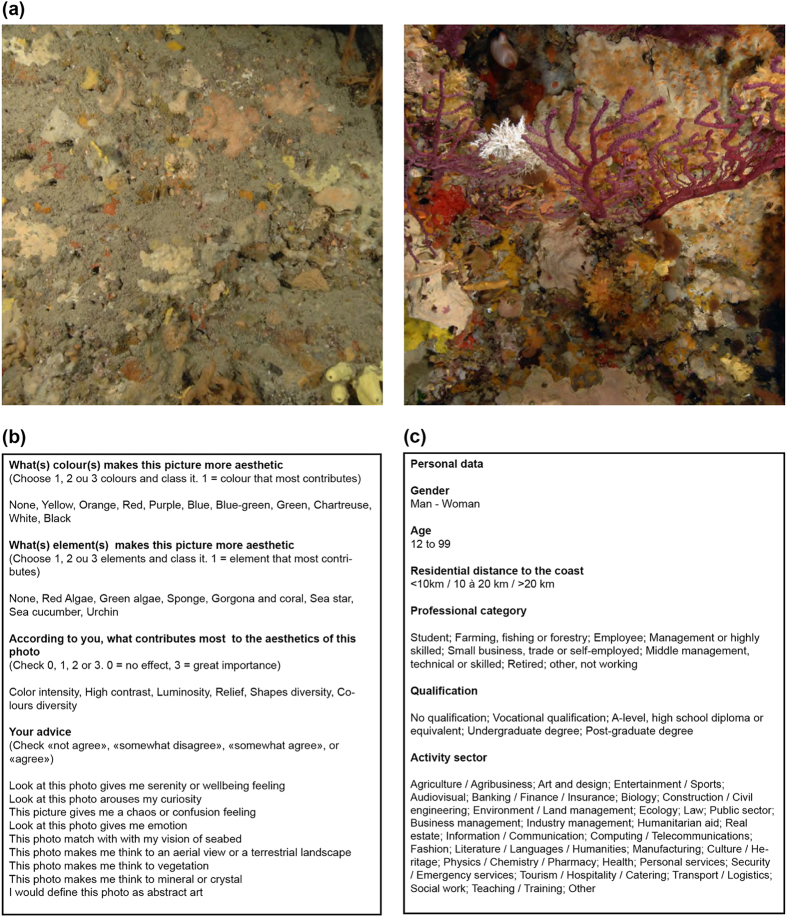
Description of the online questionnaire completed by 1260 persons. (**a**) Among the 338 photos, a randomised selection of 15 pairs of photos was presented to the observer. For each pair of photos, they had to choose the photo they felt was the most beautiful. Here we present the photos that were considered to be the least aesthetic (left) and the most aesthetic (right). Photographs taken by Florian Holon. (**b**) Among the 15 photos chosen, 3 photos were presented again and the observer had to answer questions based on semantic scales. Questions were related to visual features such as colours, colourimetry, elements or species; and their feelings and emotions experienced during the observation. **(c)** Details on their social background were also collected.

**Figure 5:**
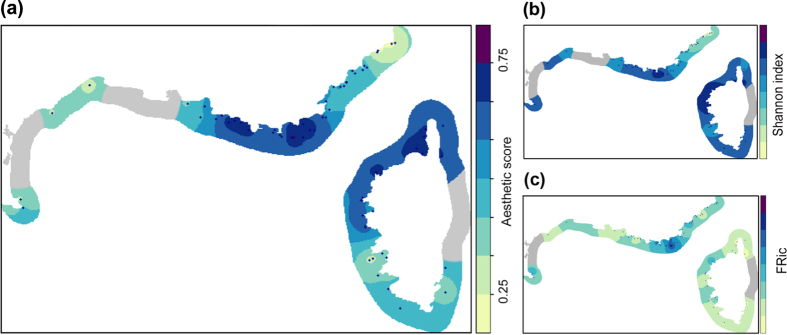
Mapping the different facets of coralligenous assemblages biodiversity along the French Mediterranean coast. (**a**) Stations (N = 113) mapped according to their aesthetic values. Each station represents the mean aesthetic score of three quadrats, scaled between 0 and 1. (**b**) Stations (N = 113) mapped according to their Shannon index values. Each station represents the mean Shannon index value of three quadrats, scaled between 0 and 1. (**c**) Stations (N = 113) mapped according to their FRic values. Each station represents the mean FRic value of three quadrats, scaled between 0 and 1. For all maps, areas were obtained using the kriging method (estimation of values between two stations).

To assess aesthetic preferences of people with regards to the coralligenous assemblages, we used an online anonymous photographic questionnaire available to the general public on a dedicated web site between April and May 2014 (1260 responses). The questionnaire consisted of a random subset of 15 pairs of photographic quadrats (among the 338 analysed ones) submitted to each participant (Fig. 4a). For each pair, the observer had to choose a ‘winner’ photo, i.e. the one they felt as the most beautiful. They then justified their choice for three randomly chosen ‘winner’ photos using a questionnaire (Fig. 4b). Information on individual social backgrounds was also collected to test the effects of socio-economic factors on aesthetic preferences (Fig. 4c).

According to the participant choices (aesthetic preferences within pairs), photos were ranked using the Elo algorithm^51^. In order to verify that the choices were not random, the distribution of the observed Elo scores (so called aesthetic scores) were compared to a random distribution (obtained by recompiling the 1260 responses with random choice of winner photos) using a Kolmogorov-Smirnov test. The effect of social background on aesthetic scores was assessed using the same methods, by comparing the whole aesthetic score distribution to aesthetic score distributions of the following categories: gender, age, professional category, sector of activity and residential distance to the coast (Fig. 4c).

### Effect of biophysical parameters on aesthetic preferences

#### Diversity and aesthetic preferences

We tested the correlation between the aesthetic score (Elo) of each photo and the different measures of biodiversity with a Spearman’s correlation test. A series of linear regressions were applied to significant correlations in order to visualize the relationship between the aesthetic score and each diversity index. As FRic and PD tend to be positively correlated with TD, we corrected this effect by correlating the residuals of linear models between TD and FRic and between TD and PD with aesthetic scores.

We tested the influence of the abiotic environment (the sum of the sediment percentage cover and the percentage of non-colonized substratum extracted after a Principal Component Analysis) on the aesthetic scores using a Spearman’s correlation test. As the abiotic environment influences biodiversity, we tested the effect of biodiversity on the residuals of the relationship between aesthetic score and abiotic environment. The same series of models described above were applied to test this relationship.

A Spearman’s correlation test was performed between the aesthetic scores of the photos and the Marginal colour heterogeneity index values for each RGB and HSV channels. Correlations between the sediment percentage cover added to the percentage of non-colonized substratum and the Marginal colour heterogeneity index values were tested using a Spearman’s correlation test. Finally, the effect of colour diversity on aesthetic scores was measured using a Spearman’s correlation test between Shannon diversity of colours among species for each quadrat and aesthetic scores.

#### Identification of key functional traits, colours and species groups

We performed a sensitivity analysis to identify which functional trait, colour and species groups (hereafter called “factor”) had the greatest impact on the relationship between the aesthetic scores and functional diversity indices. Functional indices were recalculated after removing each factor individually (Supplementary Table 1). A correlation test was then performed between these new indices (after removing each factor) and the aesthetic score. The new correlation coefficients were subtracted to the original ones (model with all traits) to measure the aesthetic importance of removed factor (δ).

To quantify the impact of species colour on the aesthetic scores, we used species coverage per photo and dominant colour (beige, black, blue, brown, green, grey, orange, pink, red, purple, white, and yellow) as defined in the literature (Supplementary Table 1). For example, we looked at the effect of the factor “red species” on aesthetic scores, by removing them from each photo and calculating a new correlation coefficient between the aesthetic score and species coverage (without red species). The same method was finally used to quantify the impact of each species group on the aesthetic scores, based on the coverage per photo of each paraphyletic group defined previously (i.e. effect of the factor “green algae” on aesthetic score).

#### Observer’s justifications

We tested whether the choice of favourite colour (Fig. 4b) was influenced by their relative dominance within the photo. For each photo, pixels hues were extracted through the R pixmap package version 0.4–11^52^. The coverage of each hue was calculated (observed coverages) as well as the frequency of preferred colours in the questionnaire (preferred frequencies) and Spearman’s correlation tests were performed for each colour between observed coverages and preferred frequencies. The favourite species were detected as for favourite colours; the same method was carried out with the observed coverages and preferred frequencies of paraphyletic groups defined previously. Finally we analysed qualitative declarations made by each observer according to ‘emotions and feelings’ experienced (Fig. 4b, ‘Your advice’). For each item, the modalities of semantic scales ranged from ‘No effect’ or ‘Totally disagree’ to ‘Great importance’ or ‘Totally agree’. We identified which justification items contributed the most to each observer’s choices by calculating the percentage of each modality per item.

#### Mapping spatial patterns of biodiversity and aesthetic value

Finally, a mean aesthetic score was calculated for each station (3 quadrats per station) and scaled between 0 and 1. To check whether diversity observed in the 3 quadrats randomly chosen for aesthetic scoring was similar to the diversity of a station (i.e. including the 30 quadrats sampled), we plotted the mean diversity per station over the 3 quadrats chosen for the aesthetic evaluation vs. the mean diversity of the 27 remaining quadrats (Supplementary Figure 5). Each station was then used to obtain a map of the distribution of an estimation of aesthetic scores along the French coast using the kriging method (estimation of values between two stations). A similar map was obtained from the mean the TD Shannon’s index, the mean FRic value and the mean PD value of each station.

All analyses were performed using R 3.2.4 (2016-03-10).

### Data availability

Relative abundances and coverages calculation are based on Doxa *et al*. 2016, available at: http://www.sciencedirect.com/science/article/pii/S1470160X1500613537. Datasets and codes used in this article are available at Dryad doi:10.5061/dryad.rv29t. Codes used for diversity indices calculation are available at: http://villeger.sebastien.free.fr/Rscripts.html.

## Additional Information

**How to cite this article**: Tribot, A.-S. *et al*. Taxonomic and functional diversity increase the aesthetic value of coralligenous reefs. *Sci. Rep.* **6**, 34229; doi: 10.1038/srep34229 (2016).

## Supplementary Material

Supplementary Information

## Figures and Tables

**Table 1:** Effect of the environment, colours heterogeneity and functional traits on aesthetic scores.

Effect of the environment and colours heterogeneity on aesthetic scores						
**(i) Aesthetic score ~ Environment variables and colour heterogeneity indices**						
Variable		Correlation with scores				
		p-value	Spearman’s rho	Bonferroni correction		
Environment variables	*% no live*	9.334 × 10^−14^	−0.452	1.886 × 10^−13^		
	*% sediment*	1.353 × 10^−11^	−0.411	2.700 × 10^−11^		
Colour heterogeneity indices	*Marginal_red*	9.680 × 10^−09^	0.353	5.808 × 10^−08^		
	*Marginal_green*	0.002	0.193	0.002		
	*Marginal_blue*	0.534	−0.039	1.000		
	*Marginal_hue*	0.611	0.032	1.000		
	*Marginal_saturation*	2.227 × 10^−16^	0.509	1.338 × 10^−15^		
	*Marginale_value*	5.940 × 10^−09^	0.358	3.564 × 10^−08^		
**(ii) Residuals (aesthetic score~environment variables) ~ Diversity indices**						
Index	Correlation with residuals					
	p-value	Spearman’s rho	Bonferroni correction			
*Shannon*	3.814 × 10^−08^	0.294	1.371 × 10^−6^			
*Simpson*	1.071 × 10^−07^	0.284	5.852 × 10^−6^			
*Nbsp*	3.394 × 10^−05^	0.223	1.220 × 10^−3^			
*PD*	1.326 × 10^−06^	0.259	4.788 × 10^−5^			
*FRic*	2.234 × 10^−05^	0.242	8.028 × 10^−4^			
**(iii) Environment variables ~ Colour heterogeneity indices**						
	% no live			% sediment		
	p-value	Spearman’s rho	Bonferroni correction	p-value	Spearman’s rho	Bonferroni correction
*Marginal_red*	0.647	−0.029	1.000	0.319	−0.063	1.000
*Marginal_green*	0.274	0.069	1.000	0.609	0.032	1.000
*Marginal_saturation*	<2 × 10^−16^	−0.508	7.200 × 10^−15^	1.49 × 10^−13^	−0.445	6.364 × 10^−12^
*Marginale_value*	0.632	−0.03	1.000	0.285	−0.067	1.000
**(b) Sensitivity analyses for functional traits**						
(i)	Trait type	Index	Correlation			
			p-value	Spearman’s rho	Bonferroni correction	
All traits	*Biological traits*	*FRic*	9.300 × 10^−10^	0.342	3.348 × 10^−08^	
	*Aesthetic traits*	*FRic*	2.480 × 10^−10^	0.353	8.928 × 10^−09^	
(ii)	Trait type	Index	Correlation			*δ*
			p-value	Spearman’s rho	Bonferroni correction	
*Aesthetic*	*Without “colour”*	*FRic*	2.009 × 10^−08^	0.315	7.236 × 10^−07^	0.037
*traits*	*Without “protect”*	*FRic*	1.114 × 10^−09^	0.341	5.572 × 10^−07^	0.012
	*Without “interest”*	*FRic*	4.063 × 10^−09^	0.330	1.461 × 10^−07^	0.023
	*Without “shape”*	*FRic*	1.296 × 10^−07^	0.298	4.680 × 10^−06^	0.055
(iii)	Trait type	Index	Correlation			*δ*
			p-value	Spearman’s rho	Bonferroni correction	
*Shape traits*	*Without “unit”*	*FRic*	5.873 × 10^−10^	0.346	2.113 × 10^−08^	0.007
	*Without “gregarious”*	*FRic*	3.886 × 10^−09^	0.330	1.401 × 10^−07^	0.023
	*Without “unit_height”*	*FRic*	3.520 × 10^−09^	0.331	1.267 × 10^−07^	0.022
	*Without “base_cover”*	*FRic*	2.820 × 10^−09^	0.333	1.015 × 10^−07^	0.020
	*Without “consistence”*	*FRic*	8.602 × 10^−09^	0.323	3.096 × 10^−07^	0.030
	*Without “base_type”*	*FRic*	2.979 × 10^−08^	0.312	1.072 × 10^−06^	0.041
(iv)						
Trait type	Trait	Correlation Abondance~Score				
		p-value	Spearman’s rho	Bonferroni correction		
*Base type*	*Encrusted*	0.032	−0.116	1.000		
	*Semi-erect*	0.026	0.345	1.000		
	*Erect*	6.822 × 10^−11^	0.629	2.455 × 10^−09^		

(a) Correlation coefficients between (i) aesthetic scores and both environment variables and colour heterogeneity indices, (ii) residuals of aesthetic score~environment variables relationship (after a Principal Component Analysis) and diversity indices and (iii) environment variables and colour heterogeneity indices. (b) Correlation coefficients between aesthetic scores and Functional Richness calculated with (i) aesthetic traits (original model) and by removing each time (ii) an aesthetic trait and (iii) a trait related to the shape. (iv) Correlation coefficients between aesthetic scores and species coverage for each base type. % no live = non-colonized substratum, % sediment = sediment percentage cover, Marginal = Marginal colour heterogeneity index, rho = Pearson’s correlation coefficient, δ = differences between original rho and new rho, Nbsp: number of species, FRic: Functional richness, PD: Phylogenetic diversity. Bold values: significant p-values. Bonferroni correction = adjusted p-values after a Bonferroni correction.
