# Supplementary material for: Taxonomic and functional diversity increase the aesthetic value of coralligenous reefs
Source: Sci Rep. 2016 Sep 28;6:34229. doi: 10.1038/srep34229 (PMC5039688; doi:10.1038/srep34229)
Supplement: Supplementary Information [file srep34229-s1.pdf]

Supplementary Information

Taxonomic and functional diversity increase the aesthetic value of coralligenous reefs

Anne-Sophie Tribot * [1,2], Nicolas Mouquet[1,2], Sébastien Villéger[2], Michel Raymond[1], Fabrice Hoff[3], Pierre Boissery[4], Florian Holon[5] and Julie Deter[1,5]

Supplementary Figure 1 Percentage of socio-economical features in the pool of persons who participated to our questionnaire (N = 1260).

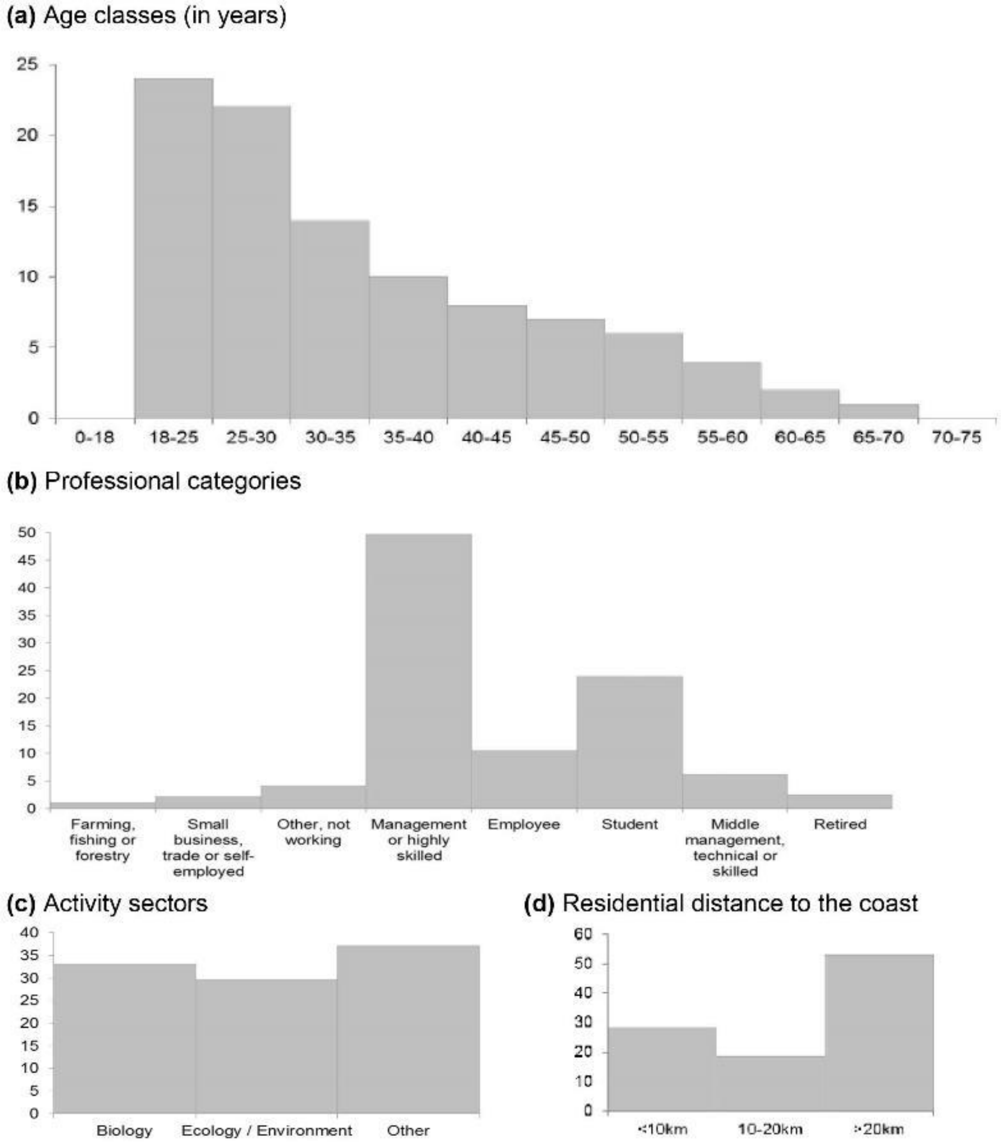

Supplementary Figure 2 Observed and random distribution of aesthetic scores. N = 2520 (2 variables x 1260 questionnaires). The horizontal axis represents the aesthetic scores of each photo: observed scores (dark grey) were calculated with the Elo algorithm based on the choices made by the 1260 participants during the online survey. Random scores (light grey) were obtained by recompiling the 1260 questionnaires with random choice of preferred picture. The vertical axis represents the number of photos for each class score. Below, the three photos from the left to the right represent: the lowest scored photo (1083), a middle score photo (1502) and the highest scored photo (1946). Photographs taken by Florian Holon.

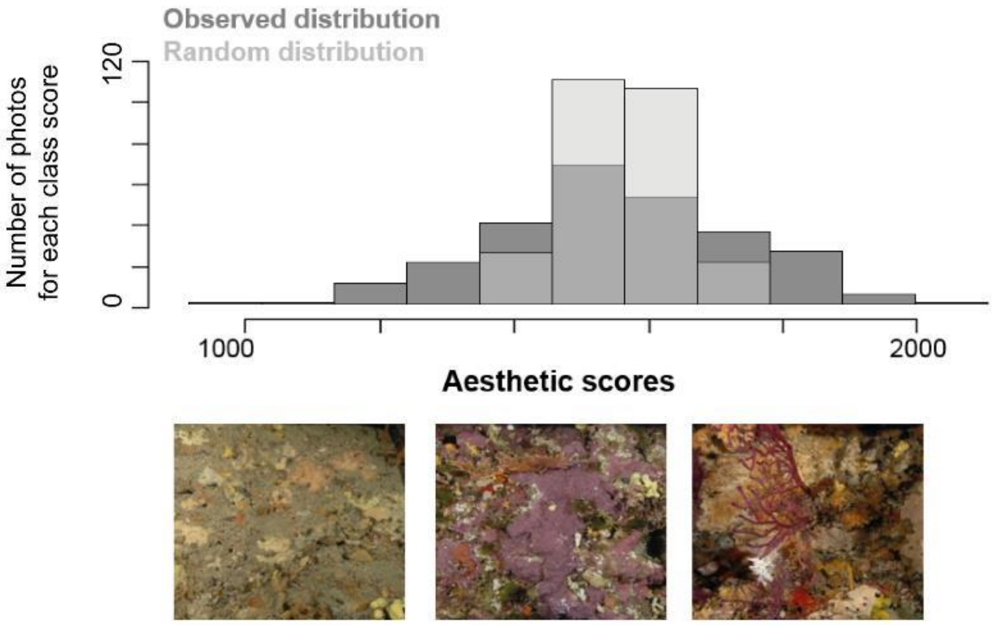

Supplementary Figure 3 (a) Sensitivity analyses of aesthetic value of paraphyletic groups according to their coverage. Code names on the vertical axis represent the species (for complete names, see Appendix 4). Grey bars on the horizontal axis represent the differences between correlations (δ) between the original model (score ~ species coverage) and the new model (score ~ species coverage after having removed the focal species). Paraphyletic groups which coverages were not correlated with aesthetic scores were not tested. N = 338 quadrats. **(b)** Species names. This table lists species used in Fig. 4 and those belonging to the paraphyletic groups that were correlated to aesthetic scores. Coralligenous species were identified using the taxonomic nomenclatures of Appeltans *et al.* (2011), Guiry & Guiry (2011) and Rodriguez-Prieto *et al.* (2013) (Doxa et al. 2015). In some cases, identification was not possible at the species level, but only the genus level (e.g. Scleractinia, Alcynonarian). Some 'Encrusting bryozoan', 'Green macroalgae' and 'Filamentous green algae' were not identified and were considered as full 'species' for further analysis. Similarly, 'Green macroalgae' and 'Filamentous green algae' refer to green macroalgae but were not further identified as identification was impossible.

(a)

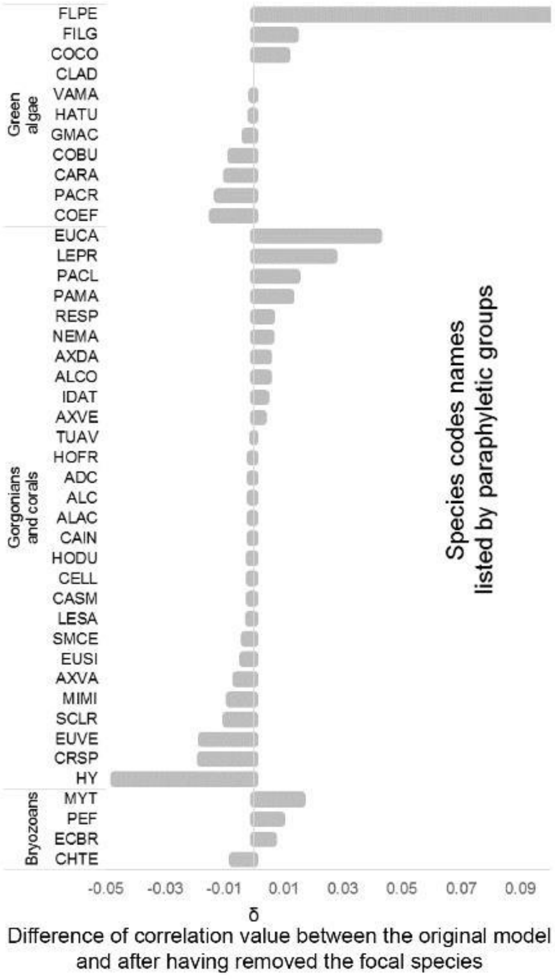

(b)

Paraphyletic group	Species name	Code
Bryozoans	"Encrusting bryozoan"	ECBR
Bryozoans	Pentapora fascialis	PEF
Bryozoans	Chartella tenella	CHTE
Bryozoans	Hydrozoa	HY
Gorgonians and corals	Crisia sp	CRSP
Gorgonians and corals	Eunicella verrucosa	EUVE
Gorgonians and corals	Scleractinia	SCLR
Gorgonians and corals	Miniacina miniacea	MIMI
Gorgonians and corals	Axinella vaceleti	AXVA
Gorgonians and corals	Eunicella singularis	EUSI
Gorgonians and corals	Smittina cervicornis	SMCE
Gorgonians and corals	Leptogorgia sarmentosa	LESA
Gorgonians and corals	Caryophyllia smithii	CASM
Gorgonians and corals	Cellaria sp	CELL
Gorgonians and corals	Hoplangia durotrix	HODU
Gorgonians and corals	Caryophyllia inornata	CAIN
Gorgonians and corals	Alcyonium acaule	ALAC
Gorgonians and corals	Alcynonarian	ALC
Gorgonians and corals	Adeonella calveti	ADC
Gorgonians and corals	Hornera frondiculata	HOFR
Gorgonians and corals	Turbicellepora avicularis	TUAV
Gorgonians and corals	Axinella verrucosa	AXVE
Gorgonians and corals	Idmidronea atlantica	IDAT
Gorgonians and corals	Axinella damicornis	AXDA
Gorgonians and corals	Alcyonium corraloides	ALCO
Gorgonians and corals	Neogoniolithon mamillosum	NEMA
Gorgonians and corals	Reteporella sp	RESP
Gorgonians and corals	Paramuricea macrospina	PAMA
Gorgonians and corals	Paramuricea clavata	PACL
Gorgonians and corals	Myriapora truncata	MYT
Gorgonians and corals	Leptopsammia pruvoti	LEPR
Gorgonians and corals	Eunicella cavolini	EUCA
Green algae	Codium effusum	COEF
Green algae	Palmophyllum crassum	PACR
Green algae	Caulerpa racemosa	CARA
Green algae	Codium bursa	COBU
Green algae	"Green macroalgae"	GMAC
Green algae	Halimeda tuna	HATU
Green algae	Valonia macrophysa	VAMA
Green algae	Cladophora sp	CLAD
Green algae	Codium coralloides	COCO
Green algae	"Filamentous green algae"	FILG
Green algae	Flabellia petiolata	FLPE

Supplementary Figure 4 Justification of observers about aesthetic preference. N = 15 items x 1 260 questionnaires.

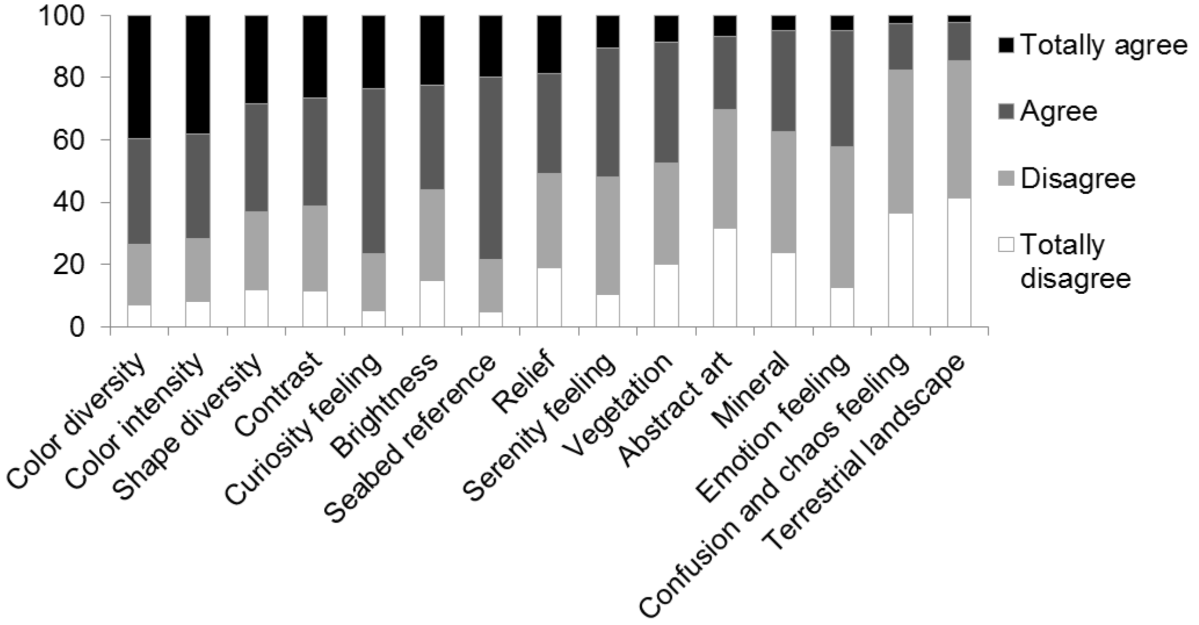

Supplementary Figure 5 (a) Correlation coefficients between the mean diversity indices for 3 quadrats and for the 27 other quadrats. Nbsp: number of species. PD: Phylogenetic diversity. FRic: Functional richness. Bold values: significant p-values. R^2 = correlation coefficient. **(b)** Relationship between the mean diversity indices for 3 quadrats and for the 27 other quadrats. (i) Linear model for the mean number of species for 27 quadrats as a function of the mean number of species for 3 quadrats. R^2 = 0.525. (ii) Linear model for the mean Shannon's index for 27 quadrats as a function of the mean Shannon's index for 3 quadrats. R^2 = 0.550. (iii) Linear model for the mean FRic for 27 quadrats as a function of the mean FRic for 3 quadrats. R^2 = 0.404. (iv) Linear model for the mean PD for 27 quadrats as a function of the PD for 3 quadrats. R^2 = 0.575.

(a)

Diversity index	Correlation between the mean for 27 quadrats and the mean for 3 quadrats			Linear model	
	p-value	Bonferroni correction	Spearman's rho	p-value	R^2
Shannon	**1.244×10^{-11}**	**4.976×10^{-11}**	0.715	**9.304×10^{-12}**	0.550
Nbsp	**$< 2.20 \times 10^{-16}$**	**8.80×10^{-16}**	0.703	**$< 2.20 \times 10^{-16}$**	0.525
PD	**$< 2.20 \times 10^{-16}$**	**8.80×10^{-17}**	0.743	**$< 2.20 \times 10^{-16}$**	0.575
FRic	**3.635×10^{-09}**	**1.346×10^{-08}**	0.632	**2.458×10^{-10}**	0.404

(b)

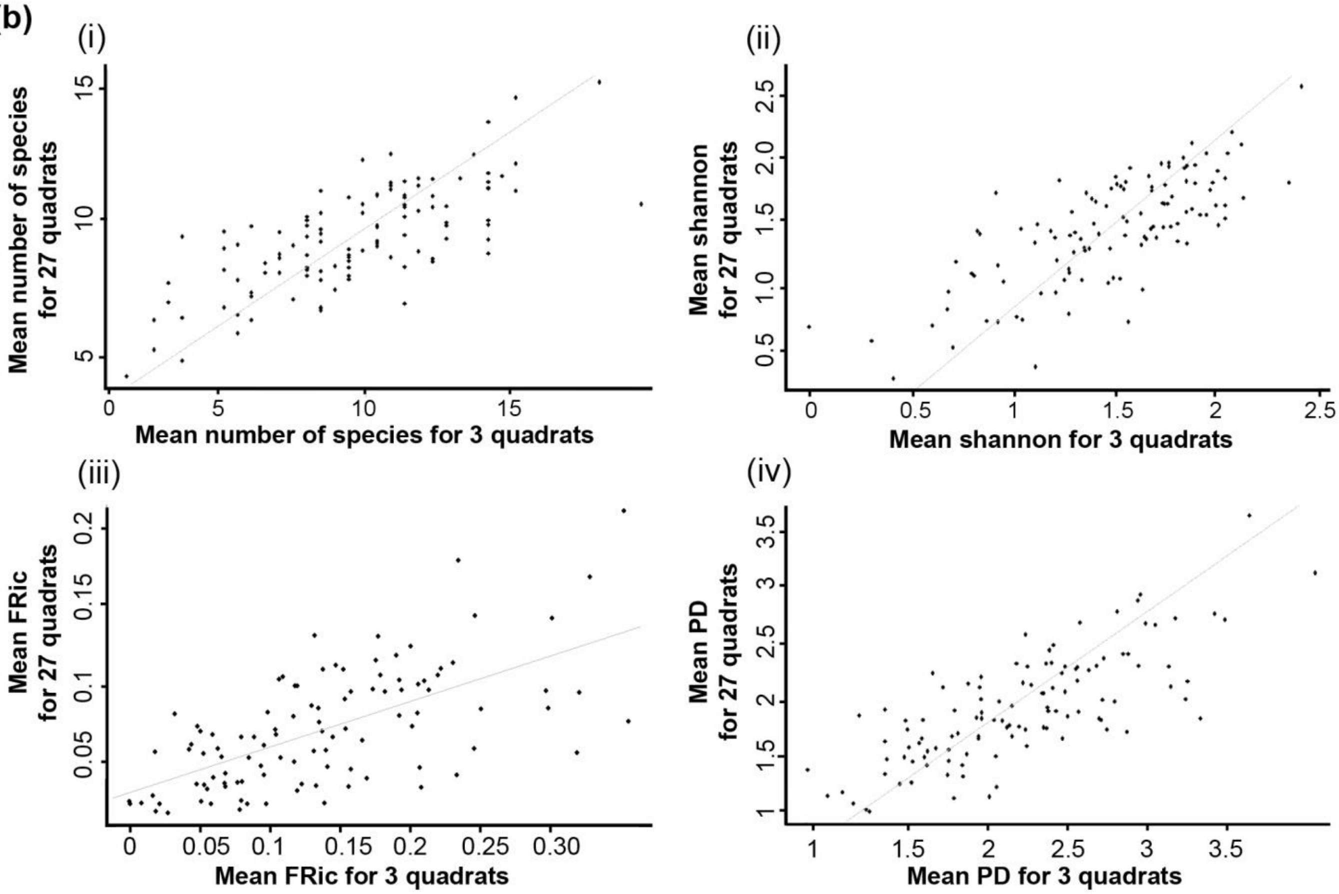

Supplementary Figure 6 Linear model for preferred paraphyletic groups frequencies for the educated professionals as a function of the non-educated professionals. Educated professionals (n = 1010): managers, engineers in "biology" and "environment" activity sectors. Non-educated professionals (n= 250): people who are neither managers nor engineers and do not work in "environment" and 'biology" sectors. P-value = 2.878 x 10^{-06}, R^2 = 0.975. Pictograms provided by DORIS http://doris.ffessm.fr.

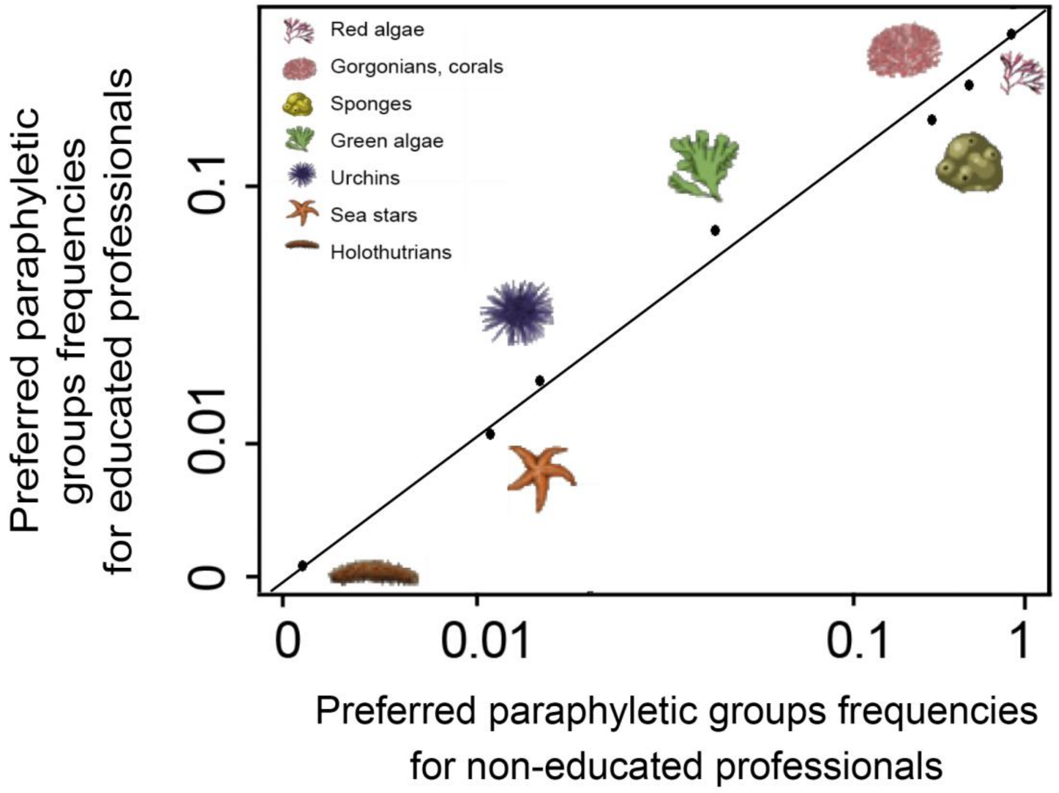

Supplementary Table 1 Functional traits assessed on all species of coralligenous assemblages. This functional database was built using information compiled from the existing bibliography (Hofrichter 2008; Commission Environnement et Biologie Subaquatiques de la FFESSM 2012; Rodriquez-Prieto et al. 2013), and in some cases the addition of an experts' judgement (Ballesteros, pers comm).

Class	Trait	Modality	Example	Description
Shape	unit	1 = individual, 2 = colony	1	what is the ecological unit
	gregarious	1 = solitary, 2 = small groups, 3 = big groups	2	how gregarious is the organism
	unit_height	mean height (in mm)	20	mean height of the ecological unit in mm
	base_cover	mean base cover (in mm)	300	cover in mm of the individual
	base_type	1= encrusting, 2 = semi-erect, 3 = erect	2	bearing of the individual or colony
	consistence	1 = soft, 2 = resistent, 3 = solid	1	consistence of the organism
Color	color_dominance	dominent color or multicolor if no dominance observed	orange	what is the dominent color of the species
	color_polymorphism	0 = no polymorphism 1 = polymorphism	3	number of possible dominent colors
	violet	0 = no, 1 = yes	0	the organism has no violet color
	blue	0 = no, 1 = yes	1	the organism has blue color
	green	0 = no, 1 = yes	1	the organism has green color
	yellow	0 = no, 1 = yes	1	etc
	orange	0 = no, 1 = yes	1	etc
	pink	0 = no, 1 = yes	1	etc
	red	0 = no, 1 = yes	1	etc
	grey	0 = no, 1 = yes	1	etc
	beige	0 = no, 1 = yes	1	etc
	brown	0 = no, 1 = yes	1	etc
	black	0 = no, 1 = yes	1	etc
	white	0 = no, 1 = yes	1	etc
	color_luminosity	1 = light, 2 = dark, 3 = multiple (light & dark)	0	Luminosity of the organism coloration
	color_tone	1 = cold, 2 = warm, 3 =multiple (cold & warm)	1	tone of the organism coloration
	transparency	0 = no (opaque), 1 = yes (translucide)	0	transparency of the organism
Reproduction	sexual_reproduction	1 = asexual, 2 = sexual, 3 = asexual and sexual	2	Type of reproduction
	reproduction_mode	1= ovipare, 2 = vivipare	2	reproductive mode
Feeding	Condition_of_food	0 = inorganic, 1 = organic alive, 2 = organic dead, 3 = organic alive and dead, 4 = inorganic and organic	1	Condition of food eaten by the species
	Size_of_food	0 = nutriments, 1 = microphagous, 2 = macrophagous	1	category of the species depending on the Size of food eaten by the species
Defense	spiked	0 = no, 1 = yes	1	spiked body
	retract	1 = no, 1 = yes	1	reacts to danger with body retraction
	stinging	2 = no, 1 = yes	1	reacts to danger with stinging
	mobile	0 = no, 1 = yes a bit, 2 = mobile	0	is the species able to move
	cryptic	0 = no, 1 = yes	0	is the species cryptic, it is difficult to see it
	shell	0 = no, 1 = yes	0	the species has a shell or a natural shelter
Interest	commercial	0 = no, 1 = yes	0	the species presents a commercial interest
	protected	1 = no, 1 = yes	1	the species is protected according to the Berne and/or Barcelone Convention
	endemic	0 = no, 1 = yes (not originar from Mediterranean sea)	0	the species is invasive
	invasive	0 = no, 1 = yes		
Ecosystem	substrate	1 = sand, 2 = living solid organisms, 3 = solid materials	23	Which substrate does the species need
	Engineering	1 = bioeroder, 2 = no ecosystem engineering, 3 = engineer	3	the species is an ecosystem engineer
	Coralligenous	0 = no, 1 = yes (coralligenous builder)	1	Is the species a coralligenous builder
Specific interaction	dominant_interaction	0 = no dominant interaction, 1 = amensalism, 2 = parasitism, 3 = predation, 4 = commensalism , 5 = symbiosis	3	Dominent type of interaction
	parasitisme	0 = no, 1 =yes	0	Parasitisme is not among the sp. interactions
	predation	0 = no, 1 =yes	1	Predation is among the sp. interactions
	commensalisme	0 = no, 1 =yes	1	Commensalism is among the sp. interactions
	symbiose	0 = no, 1 =yes	0	Symbiose is not among the sp. interactions
	competition	0 = no, 1 =yes	1	Competition is among the sp. interactions
Stress	thermoresistance	1 = cold prefered (20°C), 2 = no preference/tolerant, 3 = hot prefered or hot resistant	1	Temperature preference
	sediment	1 = no sludge tolerance, 2 = low sludge tolerance, 3 = sludge tolerance	3	Sludge tolerance
	organic_pollution	1 = no organic pollution tolerance, 2= low organic pollution, 3 = organic pollution tolerance	3	Organic polution tolerance
	salinity	1 = sea salinity prefered, 2 = no preference/tolerant	1	Salinity tolerance
	hydrodynamism	1 = calm water preference, 2 = no preference/tolerant, 3 = water current preference	3	Hydrodynamism preference
	light	1 = light preference, 2 = no preference, 3 = shadow-dark zone preference	1	Light preference

Supplementary Information references

Dramstad, W.E., Tveit M.S., Fjellstad, W.J., Fry, G.L. a 2006 Relationships between visual landscape preferences and map-based indicators of landscape structure. Landsc Urban Plan 78:465–474

Hofrichter, R. 2002 Das mittelmeer-fauna, flora, okologie band ii/1: systematischer teile (mikroorganismen, pflanzen und niedere tiere), ed. Spektrum Akademischer Verlag, 859p

Appeltans, W., Decock, W., Vanhoorne, B., Hernandez, F., Bouchet, P., Boxshall, G., Fauchald, K., Gordon, D.P., Poore, G.C.B., Van Soest, R., Stöhr, S., Walter, C., Costello, M.J. 2011 The World Register of Marine Species (WoRMS). Available from: http://www.marinespecies.org

Guiry, M.D. & Guiry, G.M. 2015 AlgaeBase. World-wide electronic publication, National University of Ireland, Galway. Available from http://www.algaebase.org

Rodríguez-Prieto, C., Ballesteros E., Boisset, F. & Afonso-Carrillo, J. 2013 Guía de las macroalgas y fanerógamas marinas del Mediterráneo occidental. Barcelona: Ediciones Omega
